# The Intriguing Landscape of Single‐Cell Protein Analysis

**DOI:** 10.1002/advs.202105932

**Published:** 2022-02-24

**Authors:** Haiyang Xie, Xianting Ding

**Affiliations:** ^1^ State Key Laboratory of Oncogenes and Related Genes Institute for Personalized Medicine School of Biomedical Engineering Shanghai Jiao Tong University Shanghai 200030 China

**Keywords:** protein analysis, single cell protein analysis, single‐cell proteomics

## Abstract

Profiling protein expression at single‐cell resolution is essential for fundamental biological research (such as cell differentiation and tumor microenvironmental examination) and clinical precision medicine where only a limited number of primary cells are permitted. With the recent advances in engineering, chemistry, and biology, single‐cell protein analysis methods are developed rapidly, which enable high‐throughput and multiplexed protein measurements in thousands of individual cells. In combination with single cell RNA sequencing and mass spectrometry, single‐cell multi‐omics analysis can simultaneously measure multiple modalities including mRNAs, proteins, and metabolites in single cells, and obtain a more comprehensive exploration of cellular signaling processes, such as DNA modifications, chromatin accessibility, protein abundance, and gene perturbation. Here, the recent progress and applications of single‐cell protein analysis technologies in the last decade are summarized. Current limitations, challenges, and possible future directions in this field are also discussed.

## Graphical abstract

In this review, recent progress and applications of single‐cell protein analysis technologies are summarized. The principles, characteristics, and applications of the emerging single‐cell multi‐omics analysis techniques are also introduced. In addition, current limitations and future prospects of single‐cell protein analysis are discussed.

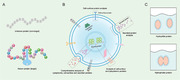

## 1 Introduction

Single‐cell analysis is uniquely capable of characterizing cell‐to‐cell heterogeneity which arises from the stochastic expression of genes, proteins, and metabolites.^[^ ^1^, ^2^ ^]^ This is particularly important for the analysis of stem cells and those cells with highly dynamic and heterogeneous nature of subpopulations, such as early embryonic development and neural stem cell differentiation. In the past decade, single cell RNA sequencing (scRNA‐seq) technology has emerged as a powerful method for the cellular heterogeneity study by determining gene regulatory networks at the whole‐genome scale.^[^ ^3^, ^4^ ^]^ However, mRNA transcript abundances only partially correlate with protein abundances,^[^ ^5^, ^6^ ^]^ typically explaining approximately one‐ to two‐thirds of the variance in steady‐state protein levels. The abundance of cellular protein is intimately linked to a remarkable series of processes, transcription, mRNA degradation, translation, and protein degradation.^[^ ^7^ ^]^ Proteins are amongst the most important molecules performing life and biological functions in living cells. Proteins and their complexes constitute the cellular cytoskeleton and structures and play key roles in most cellular processes including catalysis of biochemical reactions, transport of molecules across membranes, cell growth and division, cell adhesion, and migration. Characterizing the quantity, association, and activity of proteins is vital for understanding the molecular mechanisms of cellular processes such as cell differentiation and fate, cell signal transduction pathway, disease progression, and clinical diagnostics. To fully understand the complexity of biological processes, it is necessary to measure protein expression at the single‐cell level. However, single‐cell proteome analysis is a great challenge mainly because the protein content in a single mammalian cell is rather low,^[^ ^8^ ^]^ and proteins cannot be amplified like genes. On the other hand, large‐scale protein identification and quantification are still a challenge since the types of protein can reach over 10 000 with different expression levels in a small volume of a single cell.^[^ ^9^ ^]^

With the recent advances in engineering technology and molecular biology, there has recently been swift progress in the automation and miniaturization of single‐cell protein analysis, especially in highly multiplexed protein measurements. For example, single‐cell proteome analysis has been realized on a microfluidic chip that facilitates cell separation capture, treatment, and lysis, along with subsequent proteins and metabolites quantification. These recent breakthroughs in single‐cell proteomics methodology, such as single‐cell barcode chip (SCBC), single‐cell Western blotting (scWB), cytometry by time‐of‐flight (CyTOF), single‐cell proteomics by mass spectrometry (MS), and cellular indexing of transcriptomes and epitopes by sequencing (CITE‐Seq), give us the unprecedented opportunity to discriminate different cellular subpopulations by large scale protein profiling and infer causal mechanisms. More importantly, the trend of single‐cell analysis has transferred from “single‐omics” to “multi‐omics” (genomics, transcriptomics, proteomics, and metabolomics), making it feasible to profile chromatin accessibility, DNA, RNA, protein, and metabolites with spatiotemporal resolution. Thousands of multi‐omics quantitative data will certainly deepen our understanding of the complex relationships between transcription and translation in biological systems.

In this review, we introduced the development of single cell protein analysis methods in the past decade. We reviewed the principles and technical characteristics of existing single‐cell protein analysis methods and their applications in cell molecular biology and medical research. We also introduced the principles, characteristics, and applications of the emerging single‐cell multi‐omics analysis techniques, which can simultaneously measure DNA, RNA, proteins, and metabolites in single cells. Finally, we discussed the limitations and future challenges of single‐cell protein methods, such as low abundance proteins detection, multiplexed proteins detection, as well as the integration of large, complex, and multimodal data into specific biological models.

## 2 Overview of Current Single‐Cell Protein Analysis Methods

**Figure 1:**
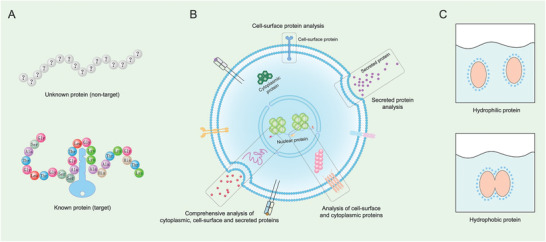
Three questions are commonly involved in single‐cell protein analysis: A) whether the proteins to be analyzed are known or unknown, B) where the proteins are inside/on‐membrane of the cell or outside the cell, and C) whether the proteins to be analyzed are hydrophobic or hydrophilic.

To better decide which pipeline is to be adopted for single‐cell protein analysis in a particular research, some basic principles need to be considered (**Figure** **1**). First, whether the proteins to be analyzed are known or unknown? This question determines the use of targeted protein detection strategy or non‐targeted detection strategy; second, whether it is necessary to acquire the location information of the proteins in the cell. For example, are we examining nuclear proteins, cytoplasmic proteins, cell surface proteins, or cell secreted proteins? It would be beneficial for selecting suitable capture and culture strategies of single cells and determining whether the cells need to be fixed and lysed. Finally, we should also understand whether the proteins to be analyzed are hydrophobic or hydrophilic. It is vital for selecting a suitable sample pretreatment system to avoid the loss of target proteins. Based on the underlying method design, current single‐cell protein analysis technology can be summarized into two major broad categories: one is targeted protein analysis technology represented by SCBC, mass cytometry, and single‐cell immunoblotting technology, and another is non‐targeted protein technology represented by MS. It is worth noting that single‐cell multi‐omics technologies have been emerged as a powerful tool to measure the genomic, transcriptomic, proteomic, and metabiotic state of individual cells. Compared with single‐cell genomics, transcriptomics, and epigenomics, multi‐omics technologies provide a wealth of multiple modalities information for researchers to unravel the complexity of biological processes in biological research. Therefore, we introduced and discussed the single‐cell multi‐omics technologies and their biological application in a separate chapter in this review.

## 3 Targeted Proteins Analysis Approaches

Most of the conventional immunoassays are antibody‐based techniques, such as WB, enzyme‐linked immunosorbent assay (ELISA), and the proximity ligation assay (PLA). Their common characteristics are having an antibody affinity reagent to identify the protein of interest and having functions of outputting different signals. However, conventional immunoassays are limited to providing quantitative detection of targeted proteins at the single‐cell level. The most straightforward solution to offer single‐cell resolution is a single‐cell microwell array, where the microfluidic chip is commonly adopted for single‐cell isolation.

### 3.1 Miniaturized Immunoassays

Most intracellular proteins are presented in low copy numbers, and therefore require analytical methods with high sensitivity and specificity. Recently, some miniaturized immunoassays methods based on microfluidic devices, such as microchip, SCBC, beads‐on‐barcode antibody microarray, microfluidic chip,^[^ ^10^, ^11^ ^]^ and array technology of single molecule^[^ ^12^, ^13^ ^]^ have been developed for multiplexed protein detection at single‐level.^[^ ^10^, ^14^, ^15^, ^16^, ^17^ ^]^

**Figure 2:**
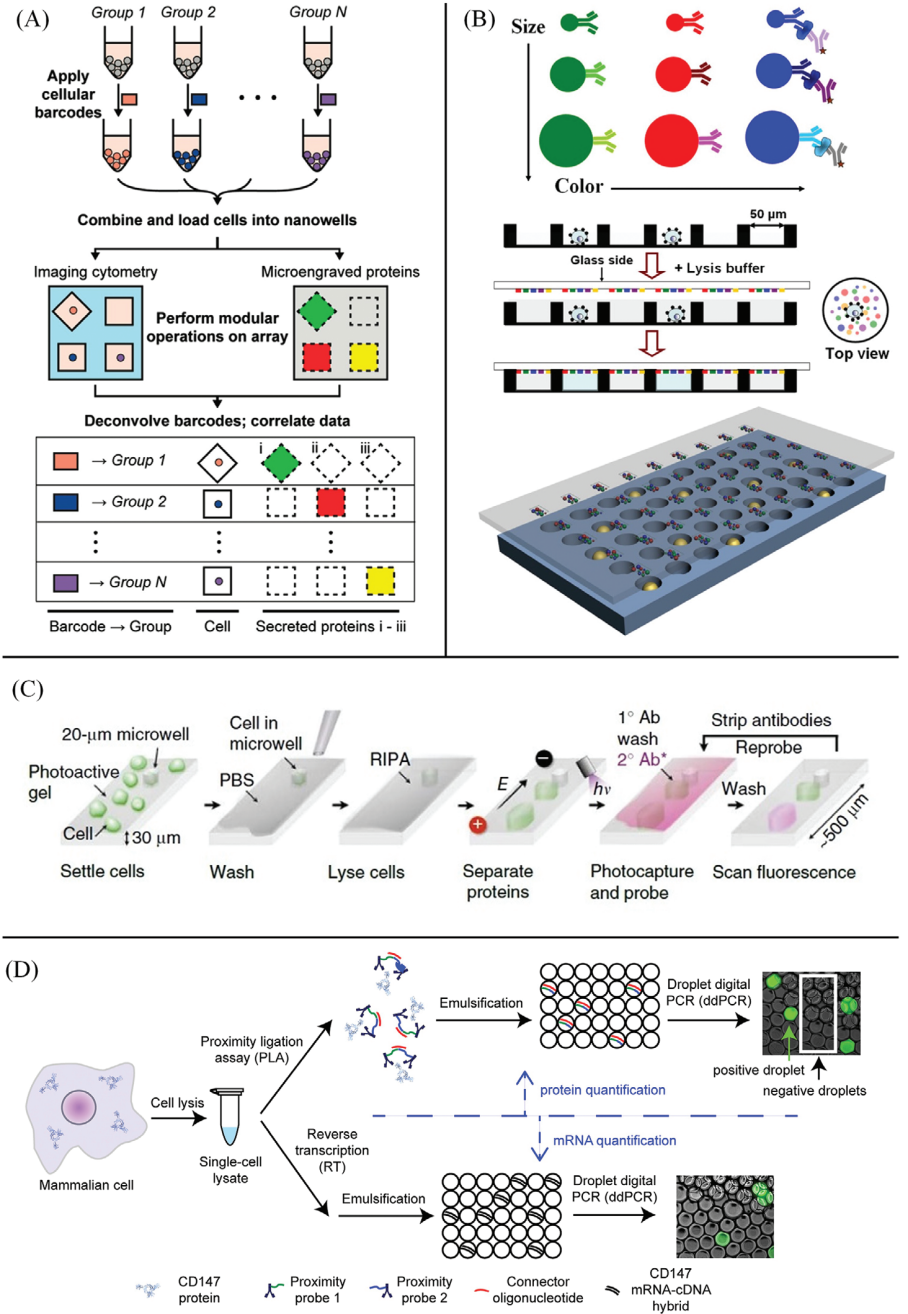
Single‐cell proteomic methods based on antibodies. A) Schematic for using cellular barcodes to increase the throughput of secretory measurements from single cells. Distinct groups of cells are labeled with unique combinations of fluorescent dyes (barcodes); the cells are combined and loaded onto the array of nanowells(top). Viability and surface marker expression (labeled on‐chip), as well as the barcodes of each cell, are determined by imaging cytometry; microengraving is performed to measure the factors secreted by the cells in each well (middle). Barcodes are deconvolved during image analysis to identify each cell's group of origin (bottom). Reproduced with permission.^[^ ^14^ ^]^ Copyright 2012, American Chemical Society. B) Schematic diagram of BOBarray‐based single‐cell multiplexed protein detection: Antibodies are conjugated to size and color‐encoded beads, and proteins are measured by sandwich‐based immunoassay (top); workflow for BOBarray‐based single‐cell multiplexed protein detection (middle). Schematic representation of glass‐bound BOBarray overlaid with an array of 60 pL sized microwells (bottom). Reproduced with permission.^[^ ^16^ ^]^ Copyright 2016, American Chemical Society. C) Principle and workflow of single‐cell western blotting: scWB analysis comprise cell settling, chemical lysis with a denaturing RIPA buffer, PAGE, UV‐initiated protein immobilization onto the gel, diffusion‐driven antibody probing, and fluorescence imaging. Reproduced with permission.^[^ ^72^ ^]^ Copyright 2014, Springer Nature. D) Workflow of simultaneous quantification of mRNA and protein in a single cell: single mammalian cells are sorted and lysed, and then the lysate is split into two fractions for protein and mRNA quantification, respectively. Protein copy number is quantified by proximity ligation assay followed by droplet digital PCR (top). mRNA copy number is quantified by reverse transcription followed by droplet digital PCR (bottom). Reproduced with permission.^[^ ^89^ ^]^ Copyright 2016, Elsevier.

The SCBC measures single‐cell proteins using an array of immobilized antibody barcode strips.^[^ ^10^, ^14^ ^]^ In an SCBC method, a single or defined number of cells is captured in nanoliter‐volume microchambers and enclosed with the antibody array. Upon lysis or secretion, proteins will bind to the array and are detected with a secondary fluorescent antibody, where the location of the fluorescence indicates the identity of the targeted protein.^[^ ^10^ ^]^ Yamanaka et al. developed a nanowell array‐based microengraving technique for quantifying the secretory responses of thousands of single cells in parallel,^[^ ^14^ ^]^ as shown in **Figure** **2**A. In the microengraving technique, single cells are isolated in a microchip array of nanowells, and a glass slide modified with cytokine‐specific antibodies is compressed on the array to capture the cytokines secreted by the cells in each well. Yang et al. described a novel SCBC method that enables profiling multiple protein markers of extraordinarily rare tumor cells at the single‐cell level.^[^ ^16^ ^]^ This SCBC method is based on a microchip consisting of 15 000 60 pL‐sized microwells and a novel beads‐on‐barcode antibody microarray, as shown in Figure 2B. Single cells were isolated in an array of 60 pL‐sized microwells for cell lysis and the subsequent detection of protein markers. The multiplexed protein detection is realized by assigning two independent identifiers of the beads, such as bead size and fluorescent color, to each protein. Moreover, the SCBC has been applied to understanding the epidermal growth factor signaling pathway, measuring T‐cell response by quantifying secreted proteins and profiling circulating tumor cells.^[^ ^16^, ^18^, ^19^, ^20^ ^]^

The basic design of the SCBC has been expanded to improve several technical aspects of the device, increasing the throughput of the SCBC to more than 1000 cells,^[^ ^21^ ^]^ expanding the multiplexing capacity up to 42 secreted proteins per cell,^[^ ^22^ ^]^ and simplifying the design to create a portable device.^[^ ^23^ ^]^ It was also modified to enable two‐cell measurements, which were used to explore how intracellular signaling depends on the distance between two cells.^[^ ^24^ ^]^ Kravchenko‐Balasha et al. showed that measurements of intracellular signaling, cellular communication, and cell distance could be integrated through a thermodynamic analysis to understand cell motility.^[^ ^25^ ^]^ Bhowmicket al. developed a portable single‐cell analysis system combining the advantages of SCBC and immunogold detection enhancement.^[^ ^26^ ^]^ The performance of this system was fully characterized by analyzing cytokine secretion of THP1 monocyte cells with a low‐end bright field microscope, indicating it did not rely on complex facilities and bulky instruments to read and record data. Nanowell‐based immunoassays have also been used to measure the secretion of multiple cytokines and to detect antibody secretion and antigen‐binding at the single‐cell resolution.^[^ ^19^, ^27^, ^28^, ^29^, ^30^, ^31^, ^32^, ^33^, ^34^, ^35^ ^]^ In miniaturized ELISAs strategies,^[^ ^15^, ^36^, ^37^ ^]^ ELISA and microfluidic devices are combined to identify intracellular proteins in single‐cell studies. Using a miniaturized ELISA, Eyer et al. quantitatively determined the concentration of the enzyme GAPDH in single U937 cells and HEK 293 cells, and the sensitivity of the system can reach attomole level per cell.^[^ ^15^ ^]^ Combined with the proteomic barcode technology and microELISA, a fully automated single‐cell proteome technology named the IsoPlexis system are developed recently.^[^ ^38^, ^39^ ^]^ IsoPlexis system adopts an SCBC and contains thousands of nanoliter‐level microchambers. Thousands of single cells are isolated in the chambers in a high‐throughput way, and then secretory proteins and intracellular proteins can be detected by surface‐immobilized antibodies. The IsoPlexis system can simultaneously analyze more than 30 secreted proteins, which has a wide application in multiplexed detection of cytokines in immunotherapy studies.

In droplet‐based microfluidics methods, individual cells in picoliter‐volume droplets are compartmentalized for high‐throughput screening and sorting of antibody‐secreting cells based on the binding or inhibitory activity of secreted antibodies, and to analyze cellular heterogeneity in cytokine‐secreting immune cells.^[^ ^40^, ^41^, ^42^, ^43^ ^]^ However, the detection results about antibody secretion and antigen‐binding in these assays are based on only endpoint data, and hardly reflect the dynamic expression of immune responses. To overcome these limitations, Eyer et al. developed a droplet‐based microfluidic technology (DropMap system) to study the humoral immune response in mice immunized with tetanus toxoid.^[^ ^44^, ^45^ ^]^ DropMap allows massively parallel kinetic analyses of single IgG‐secreting cells, with simultaneous measurement of antibody secretion rate, specificity, and affinity for the antigen. Wheeler et al. developed a new method called digital microfluidic immunocytochemistry in single cells (DISC).^[^ ^46^ ^]^ DISC allows fine time resolution and accurate dose control of the profiled stimulus, enabling the interrogation of protein phosphorylation on pulsing with stimulus for as little as 3 s.

### 3.2 Capillary Electrophoresis‐Laser‐Induced Fluorescence Detection

Although the barcoding microchip or microchamber technologies could quantify the absolute quantification of intracellular proteins, it probably exists as a false positive result because of cross‐reaction yield by limited antibody selectivity. Capillary electrophoresis‐laser‐induced fluorescence detection (CE‐LIF) is a promising technology for quantification of low copy number proteins owing to its superior high separation efficiency and the highest detection sensitivity of LIF. Xu et al. developed a novel single‐cell chemical proteomics strategy to profile low‐abundance membrane proteins in single cells.^[^ ^47^ ^]^ Shi et al. built a special LIF with a fluorescent probe to quantify low copy number intracellular proteins in a single cell.^[^ ^48^ ^]^ However, due to the vulnerability to the adsorption of proteins on the capillary wall, the detection stability is always a big challenge to the capillary‐based separation method, bringing difficulties in practical application.

### 3.3 Single‐Cell Western Blotting

WB is a robust and powerful protein analysis that combines high‐resolution gel electrophoresis and immunochemistry analysis,^[^ ^49^, ^50^ ^]^ and has the advantages of large capacity, high sensitivity, and high specificity. Therefore, it is a ubiquitous tool used widely in biological and medical research to specifically identify proteins and measure their levels, such as signaling pathways, antibody activity detection, and early diagnosis of diseases. However, conventional WB methods have some limitations including time‐consuming and labor‐intensive as most steps are done manually.^[^ ^51^ ^]^ On the flipside, the WB method typically requires micrograms of the sample, which faces the rigorous challenge in dealing with trace samples that only contain a small number of proteins. To address these problems, many miniaturized WB technologies based on capillary and microfluidic platforms have been developed in the last decade.^[^ ^52^, ^53^, ^54^, ^55^, ^56^, ^57^, ^58^, ^59^, ^60^, ^61^, ^62^, ^63^, ^64^, ^65^, ^66^, ^67^, ^68^, ^69^ ^]^ The introduction of microscale WB has significantly decreased protein separation time (within a few minutes) and sample requirements (250 ng of protein, ≈1000 cells), while enabling automatic operation, multiplexing detection, and high‐throughput capabilities.^[^ ^54^, ^55^, ^63^ ^]^ To further increase sample throughput and facilitate automation, in‐gel sieving, immobilization, and probing of separated protein have been employed in commercial instruments for eliminating the electrotransfer step in the conventional WB process.^[^ ^62^, ^70^ ^]^ In addition, the innovations of in situ immunoblotting methods have enabled microscale WB down to the single cell level,^[^ ^60^, ^71^ ^]^ providing unprecedented opportunities for studying cellular heterogeneity.

In 2014, Amy Herr et al. developed the scWB method to monitor single‐cell differentiation of rat neural stem cells and responses to mitogen stimulation.^[^ ^72^ ^]^ The principle and workflow of scWB are shown in Figure 2C. The scWB consists of a microscope slide and a thin layer of photoactive polyacrylamide (PA) gel micropatterned with microwell arrays. Cells are loaded into wells by gravitational settling are then lysed with electrophoresis buffer. Proteins in single cells are separated in the PA gel under the electric field. After separation, the proteins are immediately immobilized onto the 3D PA‐gel with UV light and consequently incubated with primary and secondary fluorescently labeled antibodies gel to detect proteins targets of interest. The sensitivity of the scWB method is comparable with that of flow cytometry (FC), and tens of proteins could be detected using stripping and reprobing, but at a relatively small dynamic range. The scWB emerged as a promising and versatile tool for the study of complex cell populations at a single‐cell resolution.^[^ ^73^, ^74^, ^75^ ^]^ More recently, Li et al. developed a single‐cell transfection analysis chip (scTAC) to rapidly and accurately evaluate transient transfection efficiency which is widely used in cellular and molecular biological studies.^[^ ^76^ ^]^ scTAC can monitor exogenous gene expression in thousands of individual host cells, enabling the acquisition of continuous protein expression even in low co‐expression scenarios. Grist et al. introduced 3D single‐cell immunoblots to detect both cytosolic and nuclear proteins from hundreds of single mammalian breast and brain tumor cells.^[^ ^77^ ^]^

While the blotted protein electropherogram of scWB retained comparable theoretical plates as microscale WB format, the overall separation had a slightly lower resolution on the chip. scWB faces major challenges in the detection of low abundance proteins and small molecular weight proteins^[^ ^78^ ^]^ because the protein band will diffuse quickly into buffer solution during the process of separation and immunoblotting. To overcome these limitations, Zhang et al. developed an enhanced single‐cell immunoblotting method by using a tetrazole‐functionalized photoactive hydrogel.^[^ ^66^ ^]^ The photoreactive tetrazole electrophilic addition reaction with a proximal nucleophile of protein can complete in a few seconds through click chemistry. Rapid and effective capture of separated protein can restrain the excessive diffusion of protein bands in the gel, and further decreases autofluorescence on the microchip. In addition, the current scWB method relies greatly on fluorescence‐based readout, and signal losses will easily occur in multiplexed detection which requires multiple rounds of antibodies stripping and reprobing. More recently, Lomeli et al. introduced scWB with a MIBI‐TOF readout by using a metal‐tagged secondary antibody, endowed the scWB method with multiplexing capability.^[^ ^79^ ^]^ Moreover, antibody incubation is one of the most critical steps in the in‐gel immunoblotting method, as the entry of antibody molecules into a wetted hydrogel is hindered by size‐exclusion, hydrophobic–hydrophobic, and electrostatic interactions. In this respect, many efforts have been made to increase the concentration of antibodies in the gels, which largely improves the antibody incubation performance in the scWB.^[^ ^80^, ^81^ ^]^

### 3.4 Proximity Ligation Assay‐Based Method

Although antibody‐based immunoassays are a powerful and widely used method, they still have limitations in detection sensitivity and specificity, and dependence upon high‐quality antibodies to recognize targeted proteins. In PLA, the paired proximity probes containing aptamer or antibody oligonucleotide complex are designed to bind to the target proteins.^[^ ^82^ ^]^ The overlapping sequences ligated upon binding protein can be subsequently extended and amplificated. In this way, the detection of a protein can be translated into the measuring output of DNA sequencing by using qPCR. Based on dual antibody recognition and powerful localized signal amplification, PLA offers increased detection sensitivity and specificity for localized detection of target proteins in cells and tissue sections.^[^ ^83^, ^84^, ^85^, ^86^ ^]^ Hu et al. reported a microfluidic digital polymerase chain reaction chip‐based PLA for the quantification of low concentration proteins.^[^ ^87^ ^]^ The limit of detection of the PLA method was achieved as low as femtomolar level with a linear dynamic range over three to four orders of magnitude. Given these advantages, PLA has the potential to overcome obstacles encountered in the detection of low abundance proteins in single‐cell analysis. PLA has been integrated with a microfluidic chip for profiling high‐content information of systematic cell signaling pathways.^[^ ^88^ ^]^ By monitoring the phosphorylation of mTOR, p70S6K, and S6 proteins, the miniaturized PLA chip resolved the dynamics of signal transduction of the Akt pathway upon PDGF stimulation at a single‐cell level.

PLA can be easily integrated into single‐cell sequencing methods, enabling multiplexed quantification of proteins and transcripts in single cells. Albayrak et al. developed a digital PLA (dPLA) method, which combines PLA and digital PCR, enabling sensitive and absolute quantification of both proteins and mRNA in single cells,^[^ ^89^ ^]^ as shown in Figure 2D. However, the large dilution factor, which is introduced by sorting and lysing individual cells in a micro‐well plate, limits the applications of dPLA for single‐cell analysis. Lin et al. developed an automated microfluidic device for dPLA measurements, which dramatically improves the sensitivity of dPLA, and with a detection efficiency of as few as 29 protein molecules per cell.^[^ ^90^ ^]^ Nolan et al. developed PLAYR (PLA for RNA)^[^ ^91^ ^]^ for highly multiplexed transcript quantification by mass cytometry, which can realize the simultaneous quantification of more than 40 different mRNAs and proteins. Furthermore, PLA and similar methods have been used to simultaneously measure mRNAs and proteins in single mammalian cells to investigate cell functions and responses.^[^ ^89^, ^90^, ^92^, ^93^, ^94^, ^95^, ^96^, ^97^ ^]^

Despite recent progress in dPLA‐based methods, some limitations hinder many applications of the current PLA method for single‐cell protein analysis. First, the aptamer screening process with SELEX (systematic evolution of ligands by exponential enrichment) is complex, and suitable antibodies with high affinity may not be available. Next, PLA needs target proteins must have two antibody binding sites for oligonucleotide overlap, but epitope availability and molecular crowding limit the antigen recognition. In addition, excessive nucleic acid probes added in the sample may raise the background, which affected the quantification of rare protein targets.

### 3.5 Micro‐Flow Cytometry

**Figure 3:**
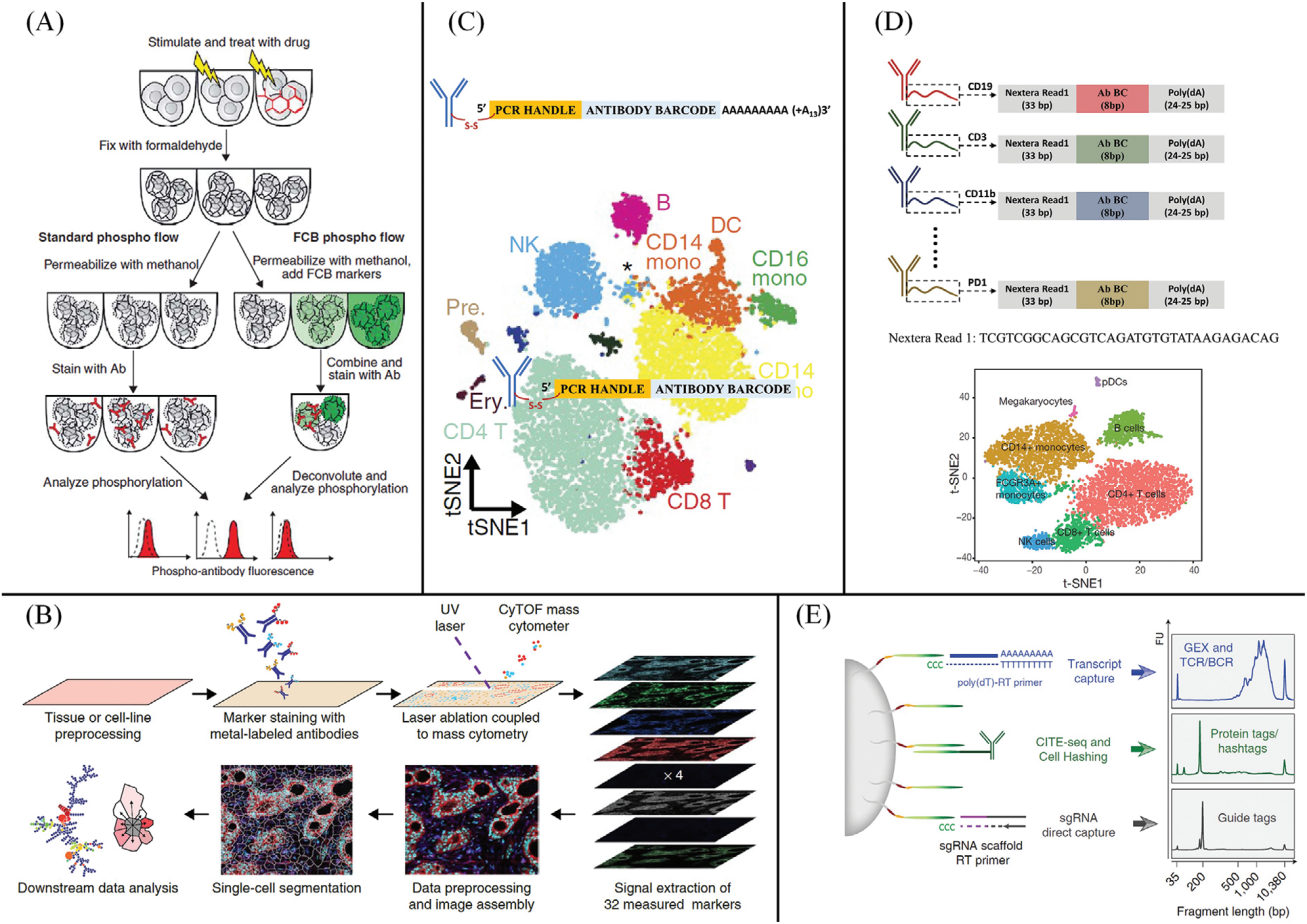
Single‐cell multiplexed protein analysis based on antibody methods. A) The principle of fluorescent cell barcoding (FCB) technique: each sample is permeabilized with methanol containing a different concentration of amine‐reactive fluorescent dyes, yielding a unique fluorescence signature for each sample. Samples are then washed, combined into one tube, and stained with antibodies. During software analysis of the acquired data, the samples are deconvoluted back to the original samples based on their FCB signature. In both standard and FCB techniques, the fluorescence of the phospho‐specific antibody in each sample is measured. In the plots, dotted lines indicate autofluorescence, and red histograms represent sample fluorescence. Reproduced with permission.^[^ ^100^ ^]^ Copyright 2006, Springer Nature. B) Workflow of imaging mass cytometry. Reproduced with permission.^[^ ^107^ ^]^ Copyright 2014, Springer Nature. C) Illustration of the DNA‐barcoded antibodies used in CITE‐seq: CITE‐seq allows detailed multimodal characterization of cord blood mononuclear cells. B, B cells; T, T cells; NK, natural killer cells; mono, monocytes; DC, dendritic cells; pre., precursors; ery., erythrocytes/blasts. Reproduced with permission.^[^ ^117^ ^]^ Copyright 2017, Springer Nature. D) Principle of REAP‐seq technique: t‐SNE visualization of eight clusters identified using the top ten significant principal components across 1664 variable genes. Cells are colored by cluster. Reproduced with permission.^[^ ^118^ ^]^ Copyright 2017, Springer Nature. E) Schematic overview of the multiple cellular modalities captured by ECCITE‐seq. ECCITE‐seq allows simultaneous detection of transcriptome, proteins, clonotypes, and CRISPR perturbations. Reproduced with permission.^[^ ^119^ ^]^ Copyright 2019, Springer Nature.

Fluorescence FC is a high throughput and multiplexing approach for profiling proteins at the single‐cell level.^[^ ^98^ ^]^ The general principles and applications of FC have been well‐reviewed by previous work.^[^ ^99^ ^]^ Cells with fluorescently labeled antibodies in a fluid stream are rapidly sorted from a mixed cell population when they pass through fluorescent activated cell sorters (FACS). In combination with fluorescent cell barcoding, as shown in **Figure** **3**A, FC allows high‐content, multiparameter analysis of single cells, making it a promising tool for basic biology and clinical research, including signaling pathway analysis, biomarker discovery, and assessment of pharmacodynamics.^[^ ^100^ ^]^ Although FC affords remarkable sensitivity, target multiplexing, and high throughput in single‐cell analysis, commercial FC instruments are bulky, costly, and require highly trained personnel for operation and maintenance. In addition, sample preparation for conventional FC requires large numbers of cells (1 × 10^6^ mL^−1^). These limitations make the conventional FC method unsuitable for applications in the situations of point‐of‐care and resource‐poor settings as well as rare samples.

Microflow cytometers that combine microfluidics and miniaturized detection systems have been developed to provide multiparameter protein analysis at a single‐cell resolution.^[^ ^101^, ^102^, ^103^ ^]^ Liu et al. developed an integrated microfluidic device named μFlowFISH for identifying bacteria in natural microbial communities.^[^ ^102^ ^]^ The μFlowFISH can perform 16S rRNA fluorescence in situ hybridization (FISH) in a hybridization chamber, where cells and probes are electrophoretically loaded, incubated, and washed for the subsequent flow cytometric detection. The μFlowFISH can monitor the numbers of *Pseudomonas* sp. with only 100–200 cells loaded into the microchip, providing an automated and sensitive platform for quantitative detection of microbial cells from complex samples. Wu et al. developed a microfluidic platform for monitoring signaling events spanning multiple time scales and cellular locations.^[^ ^103^ ^]^ The microfluidic platform seamlessly integrated two components: four vertical fluidically isolatable microchannel series for cell culture, stimulation, and preparation, two single‐cell resolution techniques on‐chip multi‐color FC and fluorescence imaging provided multiplexed and orthogonal data on cellular events. The platform provides a generic platform for profiling signaling pathways in many cell types including primary cells. In micro‐FC methods, a hydrodynamic focusing system represents the core technology of the fluidic system in a flow cytometer. In the past decades, great efforts have been made to develop highly efficient particle focusing methods using planar microfluidic approaches.^[^ ^104^, ^105^, ^106^ ^]^ Compared with previous work, stable single‐stream focusing in viscoelastic fluids is achieved at high Reynolds numbers and high flow rates, enabling accurate high‐throughput cytometry. These approaches simplify single‐stream focusing and facilitate its integration into suitable miniaturized optical systems and fluidic designs.

### 3.6 Mass Cytometry

CyTOF, also known as mass cytometry, is a creative combination of FC and MS, which enables high‐dimensional, single‐cell analysis of cell type and state.^[^ ^107^ ^]^ In CyTOF, antibodies are labeled with stable rare‐earth metal isotopes which are naturally absent in biological systems, as shown in Figure 3B. The switch from fluorescence readout to MS detection of heavy metal‐tagged antibody probes endows CyTOF with some unique advantages, such as higher multiplex detection capability, lower autofluorescence, and background noises. CyTOF allows simultaneous characterization of up to 50 cell‐surface or intracellular markers per cell.^[^ ^108^, ^109^ ^]^ In addition, with the increase of mass tags numbers, the maximum potential barcoding capability would expand exponentially. When coupled with high‐dimensional data analysis method, CyTOF enables in‐depth exploration of a signaling pathway in many biological problems, including identifying brain cell subtypes, tracking immune cell differentiation, and monitoring signal transduction through cells.^[^ ^110^, ^111^, ^112^, ^113^, ^114^, ^115^ ^]^ When coupled to a transient overexpression technique, mass cytometry‐based signaling profiling enables the assessment of how intracellular signaling states and dynamics depend on protein abundance. The recent applications of CyTOF in the single‐cell proteomic analysis have been reviewed in detail by Zhang et al. in previous work.^[^ ^116^ ^]^

### 3.7 Encoding Proteins with Oligo‐Labeled Antibodies

More recently, antibodies with DNA tags have been used to detect proteins, and the signal results are read by DNA sequencing. As the protein signal outputs can be coded by numerous specific DNA sequences, the multiplexing capacity readout for protein is nearly unlimited. For example, 10 million distinct barcodes can be generated with a 12‐mer oligonucleotide. Recently, Marlon et al. developed CITE‐Seq, which combines single‐cell RNA sequencing and multiplexed measurement of protein levels by using oligonucleotide‐labeled antibodies,^[^ ^117^ ^]^ as shown in Figure 3C. CITE‐Seq was used to simultaneously analyze the transcriptome and 13 cell surface proteins in human and mouse immune cells. The results further showed that CITE‐Seq can not only correctly identify different immune cell groups, but also help to characterize known cell subtypes. Through CITE‐Seq, they can identify the different roles of different natural killer cell subsets in the regulation of immune response under disease conditions, which cannot be detected by the single‐cell RNA‐seq method. In addition, the CITE‐seq is fully compatible with a commercially available single‐cell platform (10x Genomics) and should be readily adaptable to other droplet‐, microwell‐, and combinatorial‐indexing‐based high‐throughput single‐cell sequencing technologies.

Similarly, Peterson et al. developed the RNA expression and protein sequencing assay (REAP‐seq) to simultaneously measure gene and protein expression levels in single cells with DNA‐labeled antibodies and droplet microfluidics,^[^ ^118^ ^]^ as shown in Figure 3D. In this method, a DNA barcode of eight nucleotides was conjugated to antibodies and provided up to 65 536 unique indices. REAP‐seq was used to assess the costimulatory effects of a CD27 agonist on human CD8^+^ lymphocytes and to characterize an unknown cell type. In 2019, Mimitou et al. developed an expanded CRISPR‐compatible cellular indexing of transcriptomes and epitopes by sequencing (ECCITE‐seq)^[^ ^119^ ^]^ for the high‐throughput characterization of at least five modalities of information from every single cell, such as transcriptome, protein, clonal, and CRISPR perturbations, as shown in Figure 3E. ECCITE‐seq can recognize the differences in T cell receptor sequences, which endow specific antigens with specificity. These sequences are located at the 5′ end of an RNA molecule, which is difficult to detect by analyzing the RNA 3′ end by the scRNA‐seq method.

For CITE‐Seq and REAP‐seq, the detection of surface antigens is based on the existing FC and flow sorting antibodies. It has commercial flow reagents and has little impact on cells viability. However, if one wants to detect intracellular antigens, it is difficult to achieve antibody entry into the membrane and ensure as little leakage of the transcriptome as possible. Therefore, reagents for cell fixation and membrane breaking need to be well screened to ensure the stability of mRNA and secondary structures of intracellular proteins. Furthermore, milder permeabilization methods that allow antibodies to enter cells should be developed in the future, which may enable researchers to sequence intracellular proteins by DNA‐conjugated antibody methods.

### 3.8 In Situ Single‐Cell Proteins Analysis Methods

In situ single‐cell protein analysis is crucial for the understanding of the organization, regulation, and function of heterogeneous biological systems. Owing to the spectral overlap of commonly organic fluorophores, fluorescence microscopy allows limited multiplexed, quantitative protein analysis in a single cell. A previous study reported that up to seven fluorophores were separated by multispectral imaging.^[^ ^120^ ^]^ However, in situ profiling of many different proteins in single cells is greatly limited by the multiplexing capacity of conventional fluorescence‐imaging‐based approaches.

In recent years, technical advances in imaging mass cytometry^[^ ^107^ ^]^ and reiterative staining‐based technologies have enabled highly multiplexed protein profiling in single cells in situ. Reiterative immunofluorescence avoids the specialized and expensive instrument and can realize high sample throughput analysis. In reiterative immunofluorescence methods, antibodies labeled with different fluorophores are employed to stain their corresponding protein targets in the individual cells. Then, the cell is imaged under a fluorescent microscope to in situ quantify the abundances of the protein targets. After fluorescence imaging, the fluorescence signals of antibodies are erased to start a new immunofluorescence antibody staining and stripping cycle. In this way, reiterative immunofluorescence has the potential to quantify many different proteins targets in individual cells in situ. Gerdes et al. developed a multiplexed fluorescence microscopy method (MxIF) for quantitative, single‐cell, and subcellular characterization of multiple analytes in formalin‐fixed paraffin‐embedded tissue.^[^ ^121^ ^]^ The mild chemical inactivation of fluorescent dyes after each image acquisition round allows the reiterative immunofluorescence in iterative staining and imaging cycles. They detected 61 different protein epitopes in single‐cell staining patterns and revealed extensive tumor heterogeneity in 747 colorectal cancer subjects. However, chemical bleaching and antibody stripping often suffer from the degradation of the specimen, because chemical reagents may damage the epitopes of proteins and affect the subsequent cycles of staining. To efficiently erase the staining signals while maintaining the integrity of the antigenic epitopes, some reiterative immunofluorescence methods based on DNA strands displacement reactions have been developed.^[^ ^122^, ^123^, ^124^, ^125^, ^126^, ^127^, ^128^, ^129^, ^130^ ^]^ However, the bulky oligonucleotides can interfere with the binding affinity and specificity of the conjugated antibodies.

To address this problem, Guo et al. developed a highly multiplexed single‐cell in situ protein analysis approach with cleavable fluorescent antibodies (CFAs).^[^ ^125^ ^]^ After protein target staining, the fluorophores can be efficiently cleaved with a mild reducing reagent while maintaining antibodies' binding affinity and specificity. They labeled twelve different proteins in single cells in situ using CFAs and found the antigenicity of the protein targets was well preserved after incubation. Therefore, the CFA‐based method has the potential to quantify over 100 protein targets in single cells in situ. To further improve the sensitivity and sample throughput, Liao et al. developed a layer‐by‐layer signal amplification approach using cleavable fluorescent tyramide and off‐the‐shelf antibodies.^[^ ^130^ ^]^ In contrast to the conventional fluorescence‐imaging‐based approaches, this approach enhances the detection sensitivity reduces the imaging time by 1–2 orders of magnitude, and can be potentially applied to qualify hundreds of proteins expression in single cells at the optical resolution.

Due to the lack of signal amplification from secondary antibodies, in situ protein, immunofluorescence imaging methods often suffer from decreased sensitivity and low sample throughput. Therefore, in situ signal amplification is needed to improve sample throughput and sensitivity, especially for imaging of low‐abundance protein targets in cells. Saka et al. developed a highly sensitive in situ proteomics approach based on immunostaining with signal amplification by exchange reaction (Immuno‐SABER).^[^ ^128^, ^131^ ^]^ In Immuno‐SABER, the proteins targets are stained with their corresponding multiple DNA‐barcoded primary antibodies, which are hybridized to orthogonal single‐stranded DNA concatemers generated by primer exchange reaction. Then, many fluorescent imager oligonucleotides are hybridized to the DNA concatemers to realize signal amplification.^[^ ^131^ ^]^ Combining SABER amplification with rapid exchange cycles of fluorescent imager strands, Immuno‐SABER allows rapid spectrally unlimited multiplexing for in situ protein imaging. They demonstrated 5‐ to 180‐fold signal amplification in diverse samples, including cultured cells, cryosections, formalin‐fixed paraffin‐embedded sections, and whole‐mount tissues. More importantly, Immuno‐SABER is scalable and individually tunable for each protein target to accommodate the high dynamic range of the proteome in single cells.

## 4 Non‐Targeted Proteins Analysis Approaches

Immunostaining‐based proteomics is vitally important for high‐throughput, multiplexed protein expression profiling in different cell models. However, immunostaining‐based proteomics also has four main inadequacies: First, the selection of antigens and detection of the corresponding antibodies is based on prior immunological knowledge, and it means immunostaining‐based methods are not suitable for exploratory studies. Second, not all target‐antigens of interest can be detected because of the high dependence on commercial antibody availability. Third, it is difficult to develop a simple and accurate method for the absolute quantification of proteins because of the different antigen‐binding affinities. Additionally, the throughput and accuracy of antibody‐based methods are limited by cellular permeability, molecular crowding, epitope accessibility, and cross‐reactivity. To overcome these limitations, several single‐cell proteomics methods based on high‐resolution MS have been proposed for qualitative and quantitative analysis of thousands of proteins in single cells.^[^ ^132^, ^133^ ^]^

To enable deep, quantitative proteome profiling in MS‐based single‐cell proteomic approaches, miniaturized sample processing with high efficiency is required for nanoscale biological samples. Many efforts to profile the proteome of hundreds of cell samples based on the sample preparation method,^[^ ^134^, ^135^ ^]^ microfluidic chips,^[^ ^136^ ^]^ and the integrated proteome analysis device (iPAD),^[^ ^137^, ^138^ ^]^ have been developed in recent years. Currently, nanoliquid chromatography‐electrospray ionization‐tandem MS (nano‐LC‐ESI‐MS/MS) is one of the most sensitive MS‐based proteomics workflows and instrumentation for comprehensive characterization of complex protein samples.^[^ ^139^ ^]^ More recently, microfluidic devices and nanoscale separation techniques are employed to accompany nano‐LC‐MS for single‐cell protein quantification, which dramatically increase the efficiency of sample preparation and minimize the sample volume.^[^ ^140^, ^141^, ^142^, ^143^, ^144^, ^145^ ^]^ Among them, some promising single‐cell MS platforms such as nanodroplet processing in one pot for trace samples (nanoPOTS),^[^ ^142^ ^]^ single‐cell ProtEomics by MS (SCoPE‐MS), iPAD,^[^ ^144^ ^]^ and nanoliter‐scale oil‐air‐droplet (OAD) chip^[^ ^145^ ^]^have been developed for quantitative proteomic analysis in single cells.

### 4.1 Single‐Cell Proteome Analysis Based on Nano‐LC‐MS

**Figure 4:**
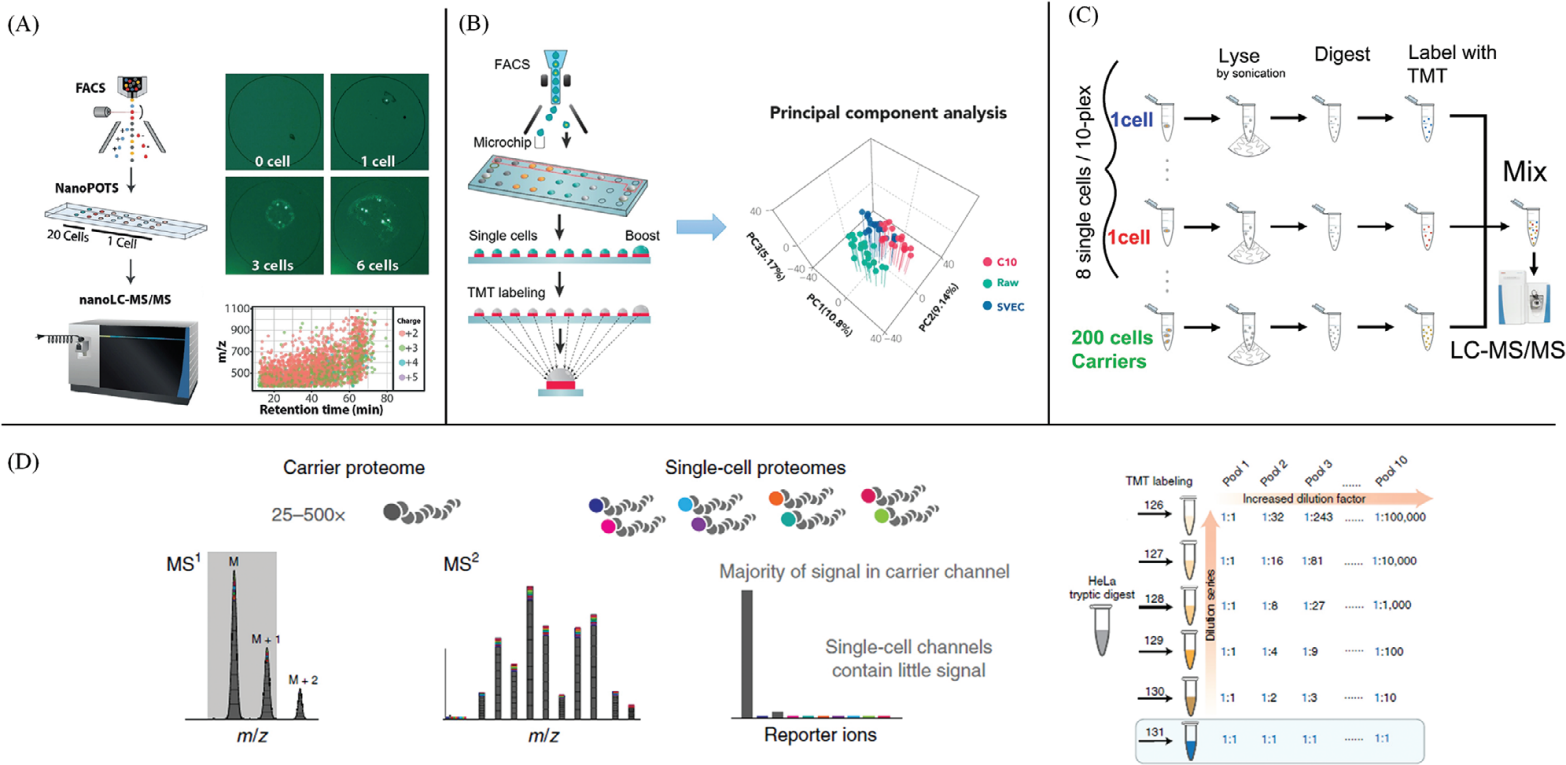
Single‐cell proteomic methods based on mass spectrometry. A) Workflow of nanobots method: Cells are FACS‐sorted into nanowells, and then cells are lysed and proteins are extracted, denatured, reduced, alkylated, and finally digested into peptides in the nanoPOTS chip. At last, Peptides are separated and sequenced with ultrasensitive nanoLC‐MS. Reproduced with permission.^[^ ^141^ ^]^ Copyright 2018, Springer Nature. B) Microfluidic nanodroplet technology is combined with tandem mass tag (TMT) isobaric labeling to significantly improve analysis throughput and proteome coverage for single mammalian cells. Isobaric labeling facilitated multiplex analysis of single cell‐sized protein quantities to a depth of ≈1600 proteins with a median CV of 10.9% and correlation coefficient of 0.98. Reproduced with permission.^[^ ^147^ ^]^ Copyright 2019, American Chemical Society. C) Conceptual diagram and workflow of SCoPE‐MS: Individually picked live cells are lysed by sonication, the proteins in the lysates are digested with trypsin, the resulting peptides are labeled with TMT labels, combined and analyzed by LC‐MS/MS. Reproduced with permission.^[^ ^151^ ^]^ Copyright 2018, Springer Nature. D) Characterization of isobaric label dynamic range and simulation of SCoPE‐MS variability: HeLa tryptic digests are labeled with TMT and pooled together in defined ratios starting from a 1:1*n* series in the first pool, up to a 1:10*n* series in the tenth pool. Observed ratios relative to the TMT131 carrier channel are calculated and log transformed. The median (dot) and median absolute deviation (MAD, whisker) of the ratio distributions of the top *n* intensity‐based peptides (*n* = 10, magenta; *n* = 100, blue; *n* = 1000, teal; all, purple) are compared against their theoretical log ratio. Reproduced with permission.^[^ ^163^ ^]^ Copyright 2021, Springer Nature.

By coupling the chip‐based nanodroplet platform to LC‐MS, Zhu et al. developed nanoPOTS to identify hundreds of protein groups from 10 to 140 cells.^[^ ^141^ ^]^ They developed a robotically addressed chip‐based nanodroplet processing platform for enhancing proteomic sample processing and analysis for small cell populations, as shown in **Figure** **4**A. With the nanoPOTS platform, the total processing volumes have been reduced from the conventional hundreds of microliters in a 0.5 mL centrifuge tube to less than 200 nL in a wall‐less glass reactor. The smaller droplet for the entire processing procedure greatly reduces sample loss during sample transfer and injection. In combination with ultrasensitive nanoLC‐MS, they validated over 3000 proteins could be identified from as few as 10 HeLa cells, which showed the potential of nanoPOTS for the analysis of proteins in single cells. Subsequently, Zhu et al. interfaced the nanoPOTS platform with fluorescence‐activated cell sorting (FACS) to realize the analysis of proteins within single mammalian cells.^[^ ^142^ ^]^ With the FACS‐nanoPOTS, about 670 protein groups were identified in single HeLa cells. By employing the MaxQuant match between runs (MBR) algorithm, FACS‐nanoPOTS significantly increased sensitivity and proteome coverage, and can detect over 1000 proteins from samples comprising subnanogram amounts of protein. Meanwhile, FACS‐nanoPOTS can differentiate cell types from enzyme‐dissociated human lung primary cells, and it can identify specific protein markers for epithelial and mesenchymal cells.

Encouragingly, with improvements in sample processing efficiency and advances in separation and MS, both label‐free and multiplexed single‐cell proteomics workflows have been benefited from the improved detection sensitivity. The nanoPOTS‐MS platform was also used to measure the progression of proteome changes during differentiation of hair cells, which enabled the de novo reconstruction of a developmental trajectory.^[^ ^146^ ^]^ Although workflow yielded 70% single‐cell proteome coverage, the analytical throughput was relatively low (at ≈8 single cells per day).^[^ ^147^ ^]^ Higher analysis throughput is required to achieve efficient analysis of a large population of cells. To solve this problem, Dou et al. combined the nanoPOTS approach with the TMT isobaric labeling method to improve analysis throughput and proteome coverage for single mammalian cells,^[^ ^147^ ^]^ as shown in Figure 4B. The nanoPOTS‐TMT platform was able to identify over 2300 proteins in 72 single cells from three murine cell populations (epithelial, immune, and endothelial cells) within 2 days. By combining nanoPOTS with nanoLC‐MS, Cong et al. achieved a cumulative gain in proteome coverage of >70% for single cells and identified 362 protein groups rather than 211 reported previously.^[^ ^148^ ^]^ To improve sample throughput and method robustness, Williams et al. designed a nanoliter‐scale autosampler and implemented it to seamlessly integrate nanoPOTS‐based sample preparation with automated LC‐MS platforms.^[^ ^149^ ^]^ To improve proteome coverage in single‐cell analysis, Kelly's group incorporated the high field asymmetric ion mobility spectrometry (FAIMS) with nanoPOTS approach, ultra‐low‐flow nanoLC, and the latest‐generation Orbitrap Eclipse Tribrid mass spectrometer.^[^ ^150^ ^]^ With the addition of FAIMS, this ultrasensitive single‐cell proteomics workflow can identify over 1000 protein groups in a single mammalian cell. The combination method greatly improved the single‐cell proteome coverage, and about 1500 proteins were additionally identified by using the MBR algorithm of MaxQuan.

To prevent protein loss and excessive dilution, Shao et al. developed a new iPAD (iPAD‐1) for single‐cell analysis.^[^ ^144^ ^]^ In the iPAD‐1, a selected single cell was directly sucked into a 22 µm i.d. capillary and then simultaneously lysed and digested. 328 proteins were identified from a single HeLa cell by their specially optimized ultrasensitive nano‐LC‐MS/MS system within 1 h. The iPAD‐1‐MS platform has some advantages including the ability to choose any living single cell in solution, an ultrasmall volume of cell treatment, nearly zero death volume in sample transmission, ultra‐high sensitivity, and fast speed. To minimum sample loss and improve sample injection efficiency for single‐cell proteomic analysis, Li et al. described a nanoliter‐scale OAD chip for achieving multistep complex sample pretreatment and injection in the shotgun mode.^[^ ^145^ ^]^ Compared to the traditional in‐tube systems, the OAD chip‐based system demonstrated three special advantages including enrichment of sequence coverage, enrichment of hydrophobic proteins, and enrichment of the enzymatic digestion efficiency.

### 4.2 Single‐Cell Proteome Analysis Based on Tandem Mass Tags

SCoPE‐MS is a recently developed method to quantify over a thousand proteins in a single cell. This method employed the strategy of two core components, such as isobaric labels and a carrier proteome, to analyze single cells.^[^ ^151^ ^]^ The stable isotope (such as ^2^H, ^13^C, ^15^N, and ^18^O) is introduced to proteomic samples, and isotope‐coded affinity tags, such as tandem mass tags (TMTs)^[^ ^152^ ^]^ have the same intact mass but contain unique mass barcodes. Then, the TMTs are detected by peptide fragmentation to enable relative quantification of proteins and digested peptides. In the SCoPE‐MS method,^[^ ^151^, ^153^, ^154^, ^155^ ^]^ the peptides from all individual cells are coded with TMTs that have the same intact mass. Therefore, peptides from all samples appear as one peak in an MS spectrum, thereby increasing the signal intensity of low‐abundance peptide ions, as shown in Figure 4C. An additional benefit of TMTs‐based method is that peptide ions can be isolated and revealed by peptide fragmentation in MS^n^ analysis,^[^ ^156^, ^157^ ^]^ as they contain unique mass barcodes. Using SCoPE‐MS, Budnik et al. quantified over a thousand proteins in differentiating mouse embryonic stem (ES) cells, and it can quantify mostly abundant proteins (over 10^5^ copies per cell) and only a few low‐abundance proteins (over 10^4^ copies per cell).^[^ ^151^ ^]^

For SCoPE‐MS, a “carrier proteome” sample (typically a mixture of cells or tissues) is spiked into single‐cell proteomes at levels ranging from 25 to 500 times to enable more peptides selection and identification.^[^ ^147^, ^151^, ^155^, ^158^, ^159^ ^]^ However, high levels of carrier proteome (>200 times) may hurt quantitative accuracy and biological conclusions. This is due to the number of ions sampled by the mass spectrometer being constrained data, any increase in the carrier proteome level necessarily decreases the number of ions sampled from the single‐cell populations.^[^ ^160^ ^]^ In addition, multiplexed proteomics data are compositional, the peptide signal in each channel needs to be converted into proportions. However, various factors, such as enzymatic digestion efficiency, post‐translational modifications (PTMs), and peptide‐to‐spectra matching uncertainty, can distort the measurements.^[^ ^161^, ^162^ ^]^ Therefore, it is necessary to examine whether high carrier proteome levels impact the dynamic range of the mass analyzer and limit the accuracy of SCoPE‐MS methods. Cheung et al. proposed the relationship between the carrier proteome amounts and evaluate quantitative accuracy, in terms of the mass analyzer dynamic range, multiplexing level, and a number of ions sampled,^[^ ^163^ ^]^ as shown in Figure 4D. They demonstrated that the increase in carrier proteome level requires a corresponding increase in the number of ions sampled to maintain quantitative accuracy. Meanwhile, they introduced a software tool named SCPCompanion to enable the rapid evaluation of single‐cell proteomic data. SCPCompanion guided experimental design and recommended data collection and data analysis parameters in SCoPE‐MS.

### 4.3 Capillary Electrophoresis‐Mass Spectrometry

CE‐MS is a proteomic technology combining electrophoretic techniques with MS to allow the rapid and efficient separation of biological molecules with nano‐ and picoliter sample volume. One of the advantages of the CE‐MS method is the ability to separate a large number of highly polar and ionic metabolites in a single cell.^[^ ^164^, ^165^, ^166^ ^]^ In addition, CE‐MS is a promising platform for peptides and proteins in whole human urine because of its multidimensional, fast, and high resolution. However, CE‐MS is not suitable for the analysis of proteins with high molecular weight (>20 kDa) because they precipitate in the acidic background electrolytes.^[^ ^167^ ^]^ Another challenge of the CE‐MS method in the single‐cell analysis is the lack of effective sample preconcentration before CE separation, leading to a lower sensitivity when compared with the LC‐MS method. To address this problem, Lombard‐Banek et al. integrated a custom‐designed single‐cell CE platform and a CE‐micro‐flow electrospray ion source (μESI) to a high‐resolution tandem mass spectrometer in a bottom‐up proteomics workflow.^[^ ^168^ ^]^ This CE‐μESI‐HRMS enabled the identification of 500–800 nonredundant protein groups by measuring as few as 0.2% of the total protein content in single blastomeres from the 16‐cell frog (*Xenopus laevis*) embryo. Compared with nano‐liquid chromatography, nano‐ESI‐HRMS can identify 1709 proteins by sequencing a larger number of peptides in single embryonic *Xenopus* cells. Through quantification of hundreds of proteins, they revealed translational differences between cells that give rise to different tissues during development. In 2019, Lombard‐Banek et al. developed a method integrating in situ subcellular capillary microsampling, one‐pot extraction and digestion of the collected proteins, peptide separation, and MS detection.^[^ ^143^ ^]^ Through their integrated design, the detection limit of the CE‐MS method for proteins reached 700 zmol (420 000 copies). They identified and quantified about 800 protein groups by analyzing just about 5 ng of protein digest (<0.05% of the total protein content) from individual cells in a 16‐cell *X. laevis* embryo. Generally, CE‐MS methods are viewed as complementary to classic MS‐based methods in the single‐cell protein and peptide analysis.

## 5 Multi‐Omics Single‐Cell Analysis

Cell activity and function are tightly regulated through the integration of signaling, epigenetic, transcriptional, and metabolic pathways. Recent advances in single‐cell genomic and proteomic technologies allow detailed measurements of RNA, mRNA, proteins, and chromatin states in single cells for phenotyping. These important developments have significantly improved our ability to understand the molecule's structure and cellular functions and how they are perturbed during physiological processes. Despite these important developments, it still lacks “multi‐omics” technologies that can effectively link cellular transcriptional states with intracellular, post‐translational states. Recent technological breakthroughs have linked single‐cell transcriptomics data with quantitative protein and metabolites measurements, mainly by qPCR^[^ ^169^, ^170^ ^]^ and microchip,^[^ ^171^ ^]^ as well as by combining FC index sorting and barcoded antibodies with sc‐RNAseq.^[^ ^94^, ^118^, ^172^, ^173^ ^]^

### 5.1 Multiplexed, Targeted Profiling of Single‐Cell Proteomes and Transcriptomes

#### 5.1.1 Single‐Cell Immuno‐Sequencing

Linking index‐sorting strategy with transcriptome measurement facilitates mapping of immune cell phenotypes onto transcriptional and intracellular protein activity and resolves some inherent limitations of only assessing transcripts. The above‐mentioned CITE‐seq and REAP‐seq convert the abundances of different cell surface proteins to DNA barcoded antibodies and enable simultaneous measurement of proteins and mRNAs in single cells.^[^ ^117^, ^118^ ^]^ By expanding the antibody barcoding strategy to 5′ capture‐based scRNA‐seq methods, ECCITE‐seq^[^ ^119^ ^]^ enables the high‐throughput characterization of six modalities of information from each single cell, including transcriptome, T‐cell receptor, surface protein, sample identity by hashtags, and sgRNA. Consequently, ECCITE‐seq could be more sensitive in detecting expression phenotypes than scRNA‐seq alone because of the high drop‐out rates of scRNA‐seq but the low drop‐out of protein detection. Mimitou et al. introduced a variant of CITE‐seq for multimodal profiling of chromatin accessibility, gene expression, and protein levels from the same cells.^[^ ^174^ ^]^ More recently, Swanson et al. developed a novel scATAC‐seq method using a droplet‐based multi‐omics platform, which simultaneously measures transcriptomics (scRNA‐seq), epitopes, and chromatin accessibility (scATAC‐seq) from thousands of single cells.^[^ ^175^ ^]^

Although the combined measurement of RNA and protein expression is realized, it still lacks suitable approaches to visualize combined transcript‐protein datasets for describing the pronounced differences in abundance and dynamic range of expression. For example, it remains unclear whether the antibody detection results of the dynamic range of protein expression are mostly in accord with that of established flow‐cytometry‐based assays in different experimental settings. Moreover, the droplet‐based scRNA‐seq method may still miss specific transcripts of interest which are lower than the detection limit.^[^ ^176^ ^]^To overcome these limitations, Mair et al. developed a targeted transcriptomics approach to simultaneously interrogate 492 immune‐related genes and 41 surface proteins that are commonly used for immunophenotyping.^[^ ^177^ ^]^ By combining high‐throughput targeted transcriptomics with oligonucleotide‐barcoded antibodies, the integrated approach was so sensitive that can detect low‐abundance transcripts while only requiring about one‐tenth of the sequencing read depth needed for the conventional scRNA‐seq method. Their study results also indicated the validation of oligonucleotide‐barcoded antibody panels is necessary for multi‐omic data interpretation. Finally, CITE‐seq and REAP‐seq are not fully compatible with intracellular proteins and have limited resolution due to the high background from the non‐specific binding of antibodies.

#### 5.1.2 Flow Cytometric Platform Combined with scRNA‐seq

**Figure 5:**
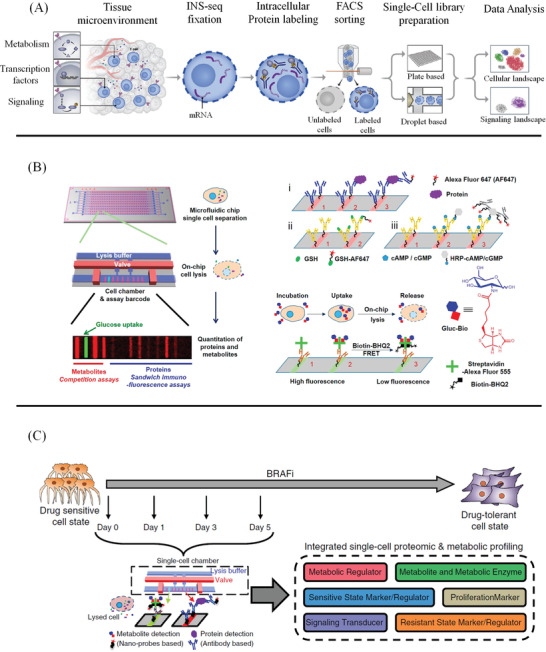
Single‐cell multi‐omics methods. A) Illustration of integrated technology for scRNA‐Seq (INs‐seq) and intracellular protein measurements: INs‐seq defines new immune subsets by TF combinations and metabolic activity. Reproduced with permission.^[^ ^184^ ^]^ Copyright 2020, Elsevier. B) Single‐cell proteomic and metabolic analysis of early drug response in M397 cells: Cells from different time points during BRAFi treatment are collected and individually analyzed using the microfluidic‐based single‐cell barcode (SCBC) technology. Each cell is characterized for the levels of six different categories of markers. Reproduced with permission.^[^ ^192^ ^]^ Copyright 2015, American Chemical Society. C) Illustration of the SCBC layout and the individual miniaturized cell chambers, and a typical fluorescence image of one set of barcodes: For proteins, antibodies are immobilized on the barcode through the DEAL method, then proteins from the cell lysate are captured by the antibody, and Alexa Fluor 647(AF647)‐labeled detection antibodies are used to generate fluorescence readout. Similarly, metabolite‐specific antibodies are immobilized, then labeled metabolites compete with those native ones from the lysed cell for the antibody binding site. For Gluc‐Bio probe detection, AF555‐labeled streptavidin is immobilized on the barcode, then Gluc‐Bio molecules from the lysed cell occupy the biotin‐binding sites of the streptavidin. The unoccupied sites are filled by Biotin‐BHQ2 molecules, the residual fluorescence positively corresponds to the amount of Gluc‐Bio molecules from the cell. Reproduced with permission.^[^ ^39^ ^]^ Copyright 2020, Springer Nature.

Recently, some novel single‐cell approaches combing flow cytometric assay and scRNA‐seq have been developed for the multi‐parametric analysis of single cells.^[^ ^178^, ^179^, ^180^, ^181^, ^182^, ^183^, ^184^, ^185^, ^186^ ^]^ FC‐based FISH (Flow‐FISH) has been undertaken to single‐cell simultaneously measurement of transcript levels and protein expression, and to gain more insight into the regulation of gene transcription and translation in individual cells.^[^ ^178^, ^179^, ^180^, ^182^ ^]^ Combining CyTOF with single‐cell transcriptomics and multiplex tissue imaging, Lavin et al. developed a multiscale immune profiling strategy to map the immune microenvironment of early lung adenocarcinoma lesions.^[^ ^181^ ^]^ Their results demonstrated that neoadjuvant immunotherapy strategies had the potential to reactivate the tumor‐infiltrating lymphocyte microenvironment and transformed tumor response to checkpoint blockade. Chen et al. developed a novel single‐cell approach protein‐indexed assay of transposase accessible chromatin with sequencing (Pi‐ATAC), which can index and quantify cell surface or intracellular protein epitopes using index FACS and can enumerate the accessible DNA elements of the same individual cell.^[^ ^183^ ^]^ Pi‐ATAC can simultaneously identify the epigenomic and proteomic heterogeneity in individual cells, which directly link the cellular phenotype and environment to the chromatin variation at the single‐cell level. Katzenelenbogen et al. developed INs‐seq, integrated technology for the massively parallel recording of scRNA‐seq and intracellular protein activity. When combined with scRNA‐seq, the INs‐seq had the potential to discover new immune subsets, by profiling intracellular and post‐translationally modified proteins (PTMs), signaling pathways, TFs, and metabolism‐related proteins,^[^ ^184^ ^]^ as shown in **Figure** **5**A.

#### 5.1.3 Microfluidic/Droplet Platform Combined with scRNA‐seq

Automated, microfluidic/droplet platforms combined with scRNA‐seq have been developed to simultaneously detect miRNAs, mRNAs, proteins, and PTMs at single‐cell resolution.^[^ ^187^, ^188^ ^]^ George et al. developed a splittable single‐cell microchip for genome‐wide transcriptome and secreted cytokine proteins in the same single cells.^[^ ^188^ ^]^ Similar strategies could also be used in the quantification of immune dynamics in single cells.^[^ ^31^ ^]^ Park et al. developed a microwell‐based platform to study patterns of gene expression, protein expression, and translation kinetics in thousands of single cells.^[^ ^189^ ^]^ Distinct patterns of transcript‐protein correlation were observed in non‐small cell lung cancer cell lines with a given protein expression. Protein‐DNA interactions play a critical role to regulate gene expression, but it remains somewhat challenging to interpret how cellular heterogeneity in protein‐DNA binding influences variability of gene expression. Rooijers et al. developed scDam&T‐seq (single‐cell DNA adenine methyltransferase identification with messenger RNA sequencing of the same cell) for the simultaneous quantification of protein‐DNA contacts and gene expression.^[^ ^190^ ^]^ Their results also revealed how genome‐lamina contacts or chromatin accessibility correlate with gene expression in individual cells.

### 5.2 Simultaneous Quantitation of Metabolites and Proteins in Single Cells

Compared to other single‐cell “‐omics” analyses, metabolomics provides a more immediate and dynamic picture of the phenotype of a cell.^[^ ^191^ ^]^ However, the metabolome is extremely difficult to measure at the single‐cell level, because the metabolic dynamics are as rapid as a few seconds. In addition, it is not possible to amplify metabolites or tag small‐molecule metabolites with fluorescent labels due to the large structural diversity of metabolites. One possible solution to cope with these problems involves a miniaturized antibody barcode microarray. The microchip contains thousands of nanowells that gently trap individual cells and culture them under well‐controlled conditions. These microchambers also allow incubation, washing, labeling, and lysis steps to be done with single cells. In this way, the lysate remains contained in the microchamber for subsequent quantification of intracellular biomolecules and metabolites.

Xue et al. developed chemical approaches for integrated metabolic and proteomic assays from single cells, by incorporating surface‐competitive binding assays and protein immunoassays onto an SCBC,^[^ ^192^ ^]^ as shown in Figure 5B. By using the SCBC, the metabolic heterogeneity and the correlative interactions between metabolites and signaling proteins can be simultaneously measured with high accuracy, providing more rich information in cellular metabolic signal regulations and their drug‐induced perturbations. Subsequently, they developed a supramolecular surface competition assay for screening of Glutamine analogs and identified new interactions between phosphoprotein signaling and cellular energy processes when integrated with SCBC.^[^ ^193^ ^]^ Zhang et al. developed a microchip‐based platform for the simultaneous detection of glucose uptake, intracellular functional proteins, and genetic mutations at the single‐cell level from rare tumor cells.^[^ ^194^ ^]^ Su et al. used microfluidic‐based SCBC technology to characterize the cellular heterogeneity of mutant melanoma cancer cells during 5 days of drug treatment, uncovering a cell‐state landscape with two paths connecting drug‐naive and drug‐tolerant states,^[^ ^39^ ^]^ as shown in Figure 5C. Xu et al. proposed a multi‐dimensional organic mass cytometry, which enabled the simultaneous analysis of six cell surface proteins and about100 metabolites (including lipids and amino acids) at the single‐cell level.^[^ ^195^ ^]^

Although swift progress has been made in the development of single‐cell multi‐omics technologies, some special considerations must be taken to apply them. One key challenge of the single‐cell multi‐omics methods is how to deal with the problem of data sparsity because the coverage of epigenome and transcriptome of individual cells remains alarmingly low. Next, it is difficult to distinguish technical noise from cell‐to‐cell variability. Therefore, optimization of experimental procedures is still needed in this respect, and fundamentally new strategies should be developed to completely overcome this limitation. Besides, current single‐cell multi‐omics methods are costly, limiting their application in large‐scale analysis of complex heterogeneous samples.

## 6 Challenges and Outlook

In the past for a long time, technology innovation in single‐cell protein analysis is relatively slow because of the lack of proper single‐cell separation and capture methods, as well as detection sensitivity. With recent advancements in the technology of microfluidics, nanotechnology, and molecular biology, single‐cell protein analysis has made a great progress in many aspects, including proteome coverage, measurement throughput, detection limitation, and quantitative accuracy. Single‐cell separation strategies include cell sorting by micropore array chip, FC, microfluidic devices, and droplet‐based methods, allowing the rapid processing of tens of thousands of cells simultaneously in a single sample. Furthermore, high‐throughput sequencing technologies provide us with large‐scale quantitative molecular datasets, which dramatically broaden the proteome‐characterization landscape. The evolution of single‐cell proteomic technologies presents its clear development track: from the characterizing of one or a few target proteins to multiplexed protein detection and multi‐omics combinative analysis in a single cell.

**Figure 6:**
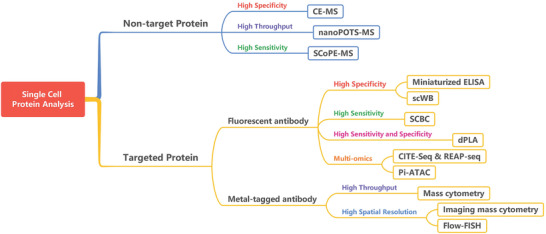
Decision tree of options for the most common different single‐cell protein analysis strategies.

Since various single‐cell proteomic methods have been used in many different biomedical research areas, researchers may often be confused about selecting a suitable method to analyze proteins in practical applications. There have been a few efforts devoted to comparing some existing single‐cell proteomic methods, however, some vital issues need to be further elucidated. Here, we outline common quantitative methods for single‐cell proteomic study and list their main technical indicators from the perspective of the detection mode, multiplicity, throughput, and sensitivity, as shown in **Table** **1**. Synthetic consideration should be taken into the selection strategy including protein physical and chemical properties, cellular localization, hydrophilicity/hydrophobic property, and sample throughput when choosing an appropriate method for single‐cell protein analysis. Thus, we further provide a decision tree to help the general reader better choose the most suitable single‐cell protein analysis method, as shown in **Figure** **6**.

**Table 1:** Overview of common single‐cell protein analysis methods

Technique	Sample	Detection method	Multiplicity	Throughput	Sensitivity	Ref.
Single‐cell barcode chip (SCBC)	Single‐cell lysis	Fluorescence detection	About 40	Medium	High	^[^ ^14^, ^15^, ^16^, ^17^, ^18^, ^19^, ^20^, ^21^, ^22^, ^23^ ^]^
Miniaturized ELISA	Single‐cell	Fluorescence detection	About 30	Medium	Medium	^[^ ^37^, ^38^, ^39^ ^]^
Droplet‐based microfluidics methods	Single‐cell	Fluorescence detection	About 10	Medium	Medium	^[^ ^40^, ^41^, ^42^, ^43^, ^44^, ^45^, ^46^ ^]^
CE‐LIF	Single‐cell	Fluorescence detection	About 10	Medium	Medium	^[^ ^47^, ^48^ ^]^
Single‐cell Western blotting (scWB)	Single‐cell lysis	Fluorescence detection	About 10	Medium	Medium	^[^ ^72^, ^73^, ^74^, ^75^, ^76^, ^77^, ^78^, ^79^, ^80^, ^81^ ^]^
Proximity ligation assay‐based method	Single‐cell	RNA sequencing	Unlimited	Medium	High	^[^ ^87^, ^88^, ^89^, ^90^, ^91^, ^92^, ^93^, ^94^, ^95^, ^96^, ^97^ ^]^
Micro‐flow cytometry	Single cells	Fluorescence detection	Up to 30	Medium	Medium	^[^ ^101^, ^102^, ^103^ ^]^
Mass cytometry	Single cells	MS detection	Up to 50	High	Medium	^[^ ^107^, ^108^, ^109^, ^110^, ^111^, ^112^, ^113^, ^114^, ^115^, ^116^ ^]^
Imaging mass cytometry	Fixed cell or tissue slides	MS detection	Up to 50	Medium	Medium	^[^ ^107^ ^]^
CITE‐Seq and REAP‐seq	Single‐cell	RNA sequencing	Unlimited	High	High	^[^ ^117^, ^118^, ^119^ ^]^
In situ protein analysis approach with cleavable fluorescent antibodies	Single cells	Fluorescence detection	Up to 100	Medium	High	^[^ ^121^, ^125^, ^128^ ^]^
Flow‐FISH	Single cells	Fluorescence detection	Up to 50	Medium	Medium	^[^ ^178^, ^179^, ^180^ ^]^
Pi‐ATAC	Single cells	RNA sequencing	Unlimited	High	High	^[^ ^183^ ^]^
NanoPOTS‐MS	Single cells	MS detection	About 1000	Medium	Medium	^[^ ^141^, ^142^, ^147^ ^]^
Nano‐LC‐MS	Single cells	MS detection	About 500	Medium	Medium	^[^ ^144^, ^145^ ^]^
SCoPE‐MS	Single cells	MS detection	About 1000	Medium	Medium	^[^ ^151^, ^163^ ^]^
CE‐MS	Single‐cell	MS detection	About 1000	Medium	Medium	^[^ ^143^, ^168^ ^]^

Despite recent developments in single‐cell proteomic technologies, challenges remain in getting a deep understanding of the complex and dynamic nature of proteome in single cells. For example, there are many complex biological factors taking place including splice variants, PTMs, large concentration dynamic range and exceedingly low amount of proteins, protein stability, transient protein associations, and dependence on the cell type or physiological state. Therefore, it is still difficult to characterize proteins selectively and sensitively in a single cell. These challenges highlight the need for more pertinent single‐cell proteomic technologies to be developed to quantify phosphorylation and other PTMs. As Harvard Medical School researcher Peter Kharchenko indicated “this would move the field beyond simple abundance‐based models to more accurate dynamic descriptions.”^[^ ^196^ ^]^ In addition, the complex and dynamic nature of proteomes dictates that there is no “one‐size‐fits‐all” proteomic strategy that can be used to address all the above‐mentioned problems. Current single‐cell proteomic methods are subject to tradeoffs between high‐sensitivity measurements and high‐throughput measurements of proteins. Before proteomic measurements, we need to make a choice between these two aspects: to measure a few proteins using an antibody‐based, sensitive approach, or to detect a lot of proteins using a highly multiplexed, unbiased approach.

Furthermore, future research needs to address some problems in sample preparation in single‐cell protein analysis. For example, efficient single‐cell separation and isolation is an especially demanding procedure, and it requires more precise handling and control of small fluid volumes for rapid transport and accurate positioning of cells. Moreover, the cellular metabolome and peptidome are variable and sensitive to sampling‐related perturbations such as temperature changes, enzyme or chemical treatments, and mechanical manipulations. It is essential to isolate individual cells from a complex environment without perturbing their contents. In addition, it is necessary to reduce intracellular biochemical activity and damage‐induced analyte loss during this process. Therefore, we need to explore some effective methods to reduce isolation‐related perturbation in the cellular proteome.

In addition, the rapid advance in single‐cell multi‐omics technologies inevitably poses many challenges for data processing and analysis. For single‐cell sequencing technologies, the integration of large, complex, and multimodal data into specific biological models and mechanisms remains a significant challenge.^[^ ^197^ ^]^ Multi‐omics show distinct advantages of simultaneously measuring multiple modalities in single cells, which enables us to get a more comprehensive characterizing of cell behavior and identity. However, it is necessary to develop appropriate strategies for tying together data across different modalities. To obtain more accurate and reliable results, it needs to properly process the massive and chaotic data before analysis to decrease bias and generate meaningful analytical results. In addition, future research needs to address some concerns in the processing of multi‐omics data, such as unifying different modalities, eliminating batch effects between experiments. Moreover, single‐cell multi‐omics methods have the computational burden of massive information from tens of thousands of cells. Future integrative analyses of single‐cell multimodal data will require more efforts in algorithm development with the incorporation of novel stochastic indexing strategies, streaming algorithms, and the development of carefully tuned high‐performance code.^[^ ^198^ ^]^

Over the past two decades, deeper insights into many cellular physiological processes have been offered by measuring the differential expression of various proteins in single cells. Each protein information provides us a snapshot through which we can partially view the dynamics of the cellular landscape. The interaction of many proteins, the activation of complex signaling pathways, and multimodal omics in single cells all together define this landscape. We believe that the solutions to these problems in the future will bring us the intriguing landscape of single‐cell protein analysis.

## Conflict of Interest

The authors declare no conflict of interest.
